# Codon-Reduced Protein Synthesis With Manipulating tRNA Components in Cell-Free System

**DOI:** 10.3389/fbioe.2022.891808

**Published:** 2022-05-13

**Authors:** Jiaojiao Li, Mengtong Tang, Hao Qi

**Affiliations:** ^1^ School of Chemical Engineering and Technology, Tianjin University, Tianjin, China; ^2^ Frontiers Science Center for Synthetic Biology (Ministry of Education), Tianjin University, Tianjin, China

**Keywords:** cell-free system, transfer RNA, codon-reduced, *in vitro*-transcription, protein synthesis

## Abstract

Manipulating transfer RNAs (tRNAs) for emancipating sense codons to simplify genetic codons in a cell-free protein synthesis (CFPS) system can offer more flexibility and controllability. Here, we provide an overview of the tRNA complement protein synthesis system construction in the tRNA-depleted Protein synthesis Using purified Recombinant Elements (PURE) system or S30 extract. These designed polypeptide coding sequences reduce the genetic codon and contain only a single tRNA corresponding to a single amino acid in this presented system. Strategies for removing tRNAs from cell lysates and synthesizing tRNAs *in vivo*/*vitro* are summarized and discussed in detail. Furthermore, we point out the trend toward a minimized genetic codon for reducing codon redundancy by manipulating tRNAs in the different proteins. It is hoped that the tRNA complement protein synthesis system can facilitate the construction of minimal cells and expand the biomedical application scope of synthetic biology.

## 1 Introduction

A codon constitutes three consecutive nucleotide bases that are recognized by a specific tRNA to integrate a single amino acid into a growing peptide chain inside the ribosome, or a stop signal that terminates protein synthesis. In general, there are 64 different combinations of the four nucleotides that encode a pool of 20 amino acids and translation stop signals in the genetic codon. Therefore, there is redundancy in the genetic codon, so that some amino acids are encoded by several so-called synonymous codons. This degeneracy of the genetic codon provides favorable conditions for us to rearrange the orthogonal sense codons (Li, 2021). As carriers of amino acids, tRNAs play a vital role in converting the genetic information from messenger RNA (mRNA) into the polypeptide chain (proteins). During protein synthesis, the aminoacylated tRNAs can bring the specific amino acid to a cognate codon of the mRNA inside the ribosome, which then elongates the polypeptide (Su et al., 2020). It is well known that different codons with their cognate tRNAs and aminoacyl-tRNA synthetases (aaRS) act orthogonally during protein translation. To reduce the redundancy of the genetic codon, it is necessary to expand the coding scope by engineering these orthogonal triplets inside a cell or *in vitro*. To establish a simplified codon set, two conditions are required in the protein synthesis reaction system: 1) the expression template is simplified to only one codon corresponding to one tRNA; 2) the orthogonality of the tRNA/aaRS/AA system needs to be maintained.

**FIGURE 1:**
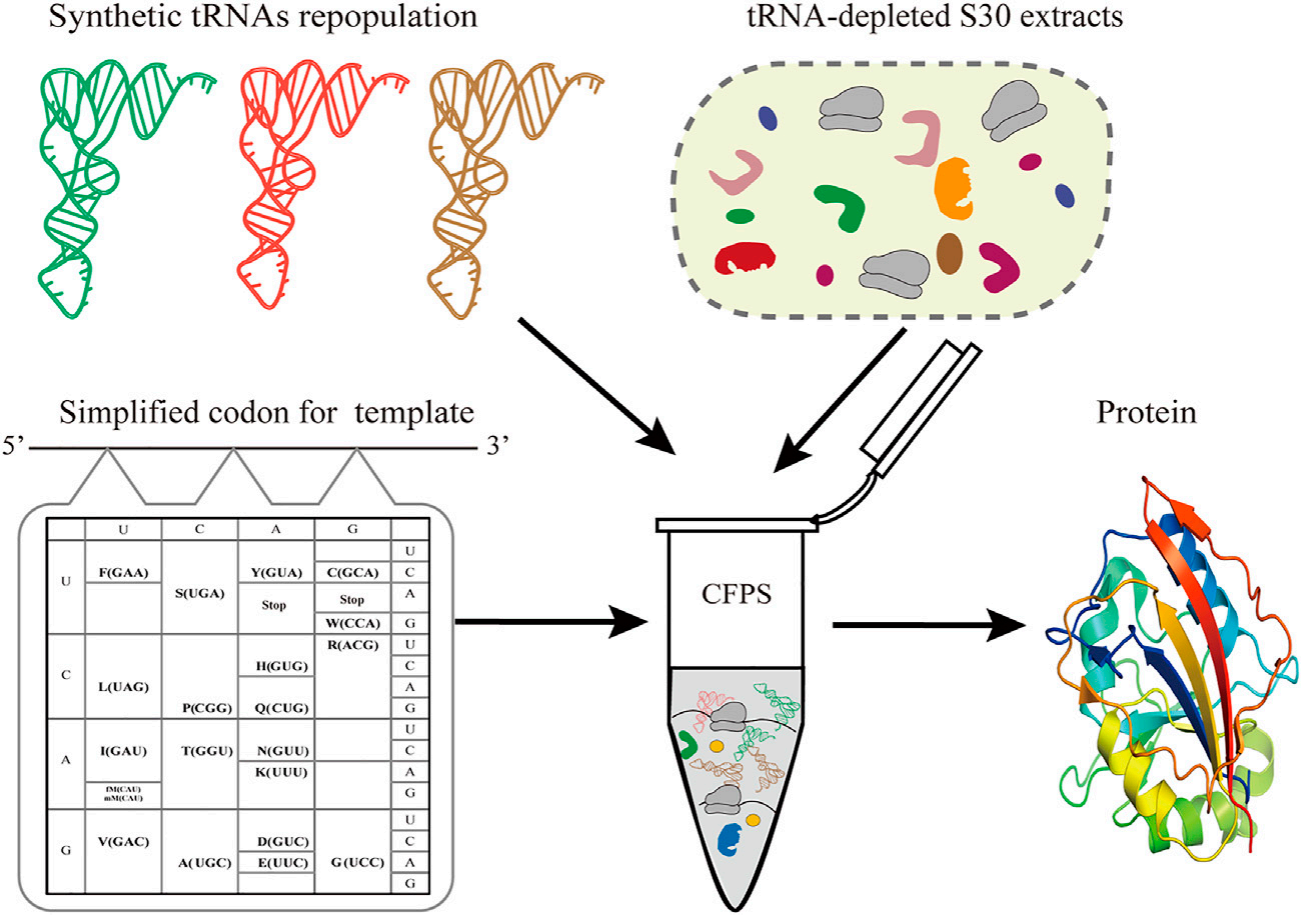
Schematic of simplified codon protein synthesis in cell-free systems. In the sfGFP template, each amino acid is encoded by one codon and simultaneously decoded by one tRNA. In a tRNA-free CFPS system, the protein synthesis of sfGFP is performed by controlling the types and concentration of synthetic tRNAs according to the designed template.

Simplified codon protein synthesis offers several profound advantages for the bioengineering and study of protein synthesis (Figure 1). First of all, the accuracy of decoding mRNA can be precisely regulated by manipulating the tRNA population added to the CFPS. This enables plasticity and flexibility of protein translation beyond codon limits or interspecies differences. Undoubtedly, this can free up more sense codons for more unnatural amino acids at multiple sites, avoiding the competition for intracellular isoacceptor tRNAs. Ideally, we only need to prepare one mRNA template to generate a variety of protein products by fine-tuning the types of added tRNAs in the CFPS.

However, reprogramming and simplifying the genetic codon is a challenging process that requires the creation of new connections between codons and amino acids. For example, strain C321. ΔA, which has a deletion of the release factor 1 (RF1) gene and is genomically recoded at all UAG stop codons to RF1-independent UAA stop codons, can be used to reassign the blank UAG codon for the incorporation of non-canonical amino acids (ncAAs), which is favorable for industrial protein production (Lajoie et al., 2013). To reduce competition effects in living cells when expanding the genetic codon, it is necessary to use the multiplex genome editing approach, which is time-consuming and labor-intensive, while only one codon is released. Moreover, due to the existence of the wobble base pair, effective decoding by native tRNAs is limited. In one study, Phe and naphthylalanine were respectively assigned at the UUC and UUU codons in a Phe-auxotrophic *Escherichia coli* (*E. coli*) strain (Link and Tirrell, 2005). When using the heterologous tRNA/aaRS/ncAAs system, there is a 20% false incorporation rate in decoding that results from some wobble effects that do not follow the Watson-Crick base pair rules (Kwon et al., 2003). The feasibility of the strategy above depends on the deletion of competing host tRNA(s). Some practical approaches arose when considering strategies to increase the reassignment efficiency for removing the native tRNAs in *E. coli*. It has been proved that deletion of a competing endogenous Arg-tRNA by genetic manipulation and complementation in *E. coli* could successfully reassign the rare AGG (Arg) codon to different ncAAs (Lee et al., 2015; Mukai et al., 2015). However, the entire genome of the cell needs to be re-synthesized, including the knock-out of tRNA genes with sense codons, while the removal of redundant tRNAs may reduce the viability of the cells, leading to a decreased protein production capacity. In addition, it is unwise to engineer the edition and activation sites of the two aaRS, since this requires large mutant libraries and tedious screening. Due to the precise regulation of the native translation system and the high cost of genome reassignment, it is difficult to simplify the codon table and reassign blank codons to new amino acids used in protein synthesis with the simplest codon form.

**FIGURE 2:**
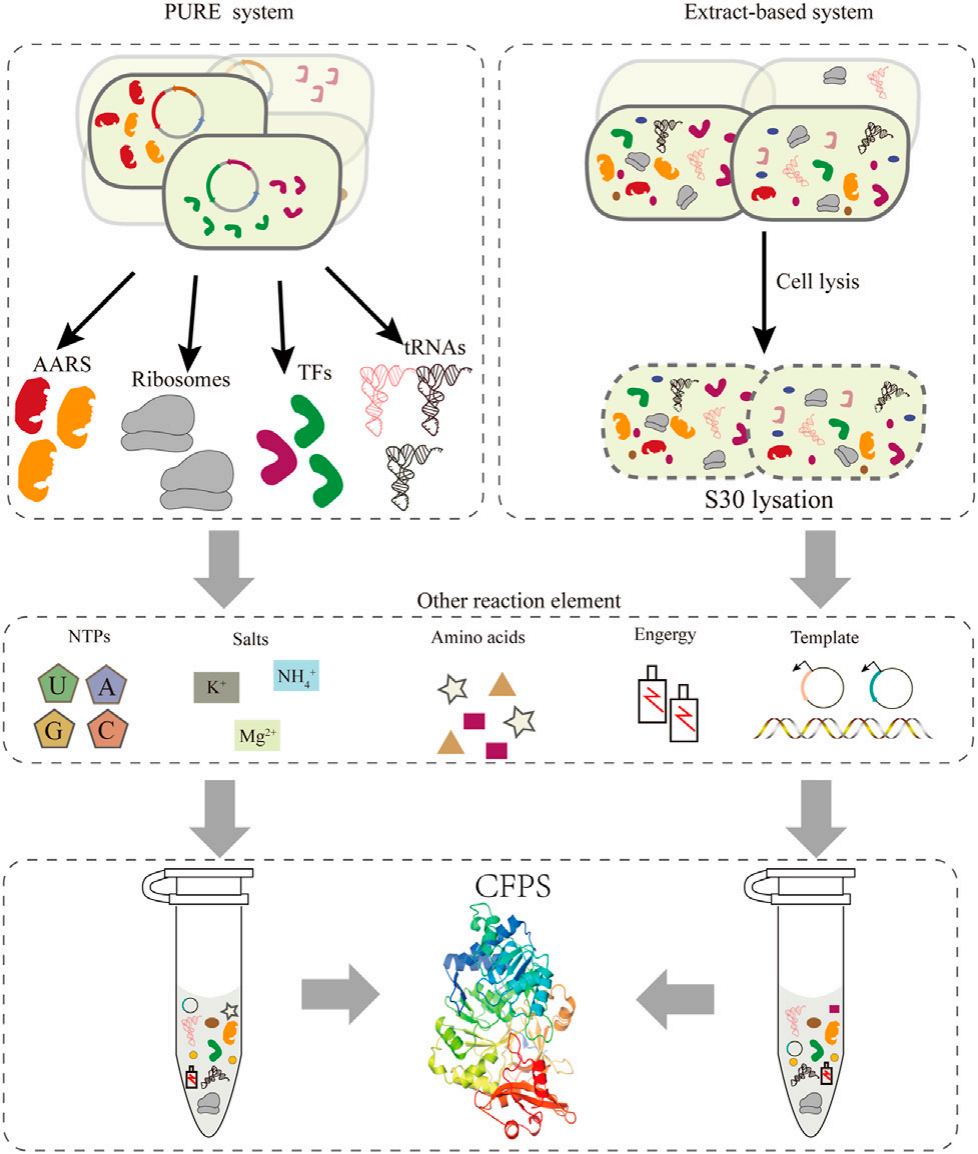
Two strategies exist for implementing CFPS: the PURE system and the extract-based system. In the PURE system (left), each essential factor associated with protein translation is purified separately from overexpressing cells, including aaRS, ribosomes, translation factors, tRNAs, etc. In addition, the corresponding template and reaction buffer components are added to construct a functional translation system. The extract-based system (right) is simple and rapid, since it relies on preparing the native cellular extract by lysing cells.

Compared to the bottleneck problems of *in vivo* systems, an alternative approach is cell-free protein synthesis (CFPS), wherein the components related to transcription and translation can be handled easily according to the experimental requirements. In the last 2 decades, the CFPS system has been developed into a promising technology and established a new field of protein synthesis beyond cells (Hodgman and Jewett, 2012; Lu, 2017). Moreover, it is an effective approach for the extensive reassignment of sense codons *in vitro*. Recently, cell-free gene expression has shown advantages in the production of membrane proteins (Schoborg et al., 2018), therapeutic proteins (Lu et al., 2014; Min et al., 2016), natural products (Li et al., 2018; Moore, 2019), and other value-added chemicals (Yang et al., 2021), and many emerging applications (Liu et al., 2019). Compared with the intact cell system, CFPS generally produces higher yields and breaks the limitation of the cell by providing more energy for protein synthesis. At present, cell-free systems can be divided into two major classes (Figure 2). One relies on protein synthesis using purified recombinant elements, called the PURE system, which can be reconstructed easily by purifying all translation factors *via* histidine (His)-tags (Shimizu et al., 2001). This system allows the control of the concentrations of all the translation elements, offering greater flexibility for protein synthesis (Shimizu et al., 2005). However, its high cost of purification and concentration-tuning work compared with the extraction system impeded its broader application. Two approaches to improve the scalability of the PURE system were one-pot nickel nitrilotriacetic acid (Ni-NTA) purifications, which use a single batch culture at low cost (Lavickova and Maerkl, 2019), and PURE 3.0 consisting of three high-copy expression plasmids, which enables the bulk purification of necessary translation factors (Shepherd et al., 2017). Extract-based systems, which provide the whole translation machinery in a cell lysate, are another class of CFPS. Extract-based systems are simple and rapid, while also containing factors that contribute to correct folding of functional proteins, such as glycosyltransferases (Jaroentomeechai et al., 2018; Kightlinger et al., 2018). Taken together, CFPS as an open platform in which almost any molecule in the reaction system can be controlled subtly for different experimental purposes. A promising application of this approach is editing of the genetic codon and reassigning sense codons for new information. Exerting better control over the cell-free system entails extract processing and the selective depletion of components of the translation machinery. However, it is necessary to include effective measures to delete the native total tRNA or individual specific tRNAs in this cell-free system to preventing decoding errors.

Here, we provide an overview of the emerging methods for removing the redundancy of the genetic codon to synthesize functional proteins based on a reduced set of tRNAs in the cell-free system. Firstly, we review the published tRNA-depleted S30 extracts including the depletion of total tRNA and specific tRNAs. Additionally, we summarize emerging methods for the purification of specific individual tRNAs *in vivo* and *in vitro*. Finally, we focus on the different reduced codons of different protein syntheses in the PURE system without total tRNA or the tRNA-depleted S30 extract system. More importantly, we point out current trends in the development of minimal codon protein synthesis systems for synthetic biology, biotechnology, and other potential fields.

## 2 tRNA-Depleted S30 Extracts

**FIGURE 3:**
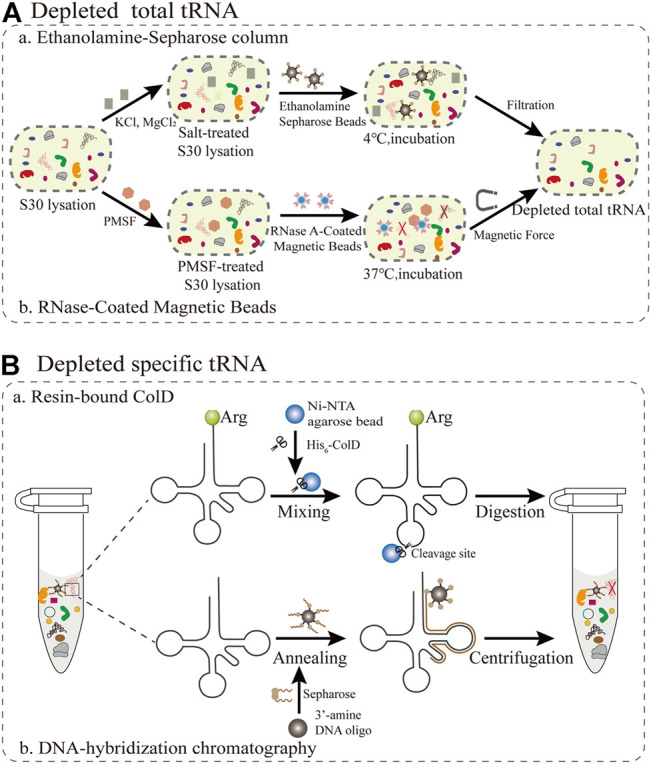
Emerging methods for removing all or individual tRNAs during S30 extraction **(A)** The removal of total tRNAs from S30 extracts based on ethanolamine agarose and RNase-Coated Magnetic Beads **(B)** Resin-bound ColD and DNA-hybridization chromatography for the removal of specific tRNAs.

As adapter molecules, tRNAs play a key role in converting the genetic codon information to the corresponding amino acid sequence in the growing polypeptide chain. Since the molecular weights of tRNAs and small molecules are relatively close, direct dialysis is not suitable for the removal of tRNAs. The main obstacle is the competition between synthetic and endogenous tRNAs, resulting in the production of truncated or inactive proteins. Theoretically, this competition can be averted as long as native tRNAs are eliminated from the cell-free system. Therefore, the target protein can be synthesized intrinsically in a one-to-one relationship among tRNAs, amino acids, and codons. Because all components of the PURE system, such as total tRNA and translation cofactors, are controllable and commercially available, it is simple and convenient to inject single tRNAs (Tuckey et al., 2014). In this section, we mainly summarize and focus on the current methods for removing total tRNAs and specific tRNAs in S30 extraction (Figure 3).

### 2.1 Depletion of Total tRNA

#### 2.1.1 Ethanolamine-Sepharose Column

The first reported method for the removal of total tRNA from S30 extracts is based on ethanolamine agarose (Figure 3A). It was discovered by accident that 90% of native tRNA in rabbit reticulocyte lysates could be separated by covalent interactions using the chemical groups of ethanolamine anchored to the resin. (Jackson et al., 2001). For this method, a column of epoxy-activated Sepharose^®^ 6B is more sensitive than ω-aminododecyl-Sepharose. This lysate system depends on supplementation with tRNA and is only applicable for the synthesis of smaller and medium-size proteins, but not for large proteins. The ethanolamine-Sepharose column procedure can expand opportunities for manipulating the tRNA population for reorganizing the genetic codon. However, this method is not universally applicable for all extracts. The most critical step is to optimize the concentration of different salts in the reaction, which influence the interaction between the tRNAs and the ethanolamine-Sepharose resin. Furthermore, there are still several challenges redesigning sense codon for ncAAs owing to the residual total tRNAs. Subsequently, the buffer used to equilibrate the resin was substituted with pure water, resulting in the elimination of about 95% of the total endogenous tRNAs in *E. coli* extracts (Ahn et al., 2006). Although this treatment process is simple and the removal efficiency is improved, a small amount of tRNA is still present, which increases the risk of residual tRNA coupling with amino acids. To alleviate the negative effects, reaction conditions including salts, ionic strength, temperature and retention time could be optimized to increase the interaction between tRNA and ethanolamine agarose so as to facilitate the construction of a tRNA complement protein synthesis system.

#### 2.1.2 RNase-Coated Magnetic Beads

The reassignment for sense codons requires the complete removal of native tRNAs from the cell extract. A promising emerging approach is based on RNase-coated magnetic beads and a phenylmethylsulfonyl fluoride (PMSF)-treated cell extract, which results in near-complete tRNA depletion (Salehi et al., 2017). For this approach (Figure 3A), ribonuclease A (RNase A) attached to superparamagnetic beads was more convenient than an immobilized RNase A Resin in controlling the RNase degradation ability and subsequently removing the RNase from the extract (Kunda et al., 2000). In addition, this method can make full use of the protective effect of nucleoproteins, so that it not only degrades tRNA but also ensures the activity of rRNA (Suhasini and Sirdeshmukh, 2006). PMSF treatment has a two-sided effect, since it inhibits proteases fivefold less than using RNase inhibitor, while also preventing RNaseA from leaching into the cell extract. Notably, the rate of tRNA removal was directly measured using quantitative real-time PCR (qPCR) with tRNA-specific primers (Kralik and Ricchi, 2017). The average removal ratio for all assessed native tRNAs was 99.3% following RNase A treatment for 60 min. However, there are still some problems that need to be addressed. The cell extract treated with RNase A-beads was able to produce a designer peptide containing 40 Val residues with the addition of a synthetic tRNA, but it remains unclear if proteins with larger molecular weights can be synthesized. The removal of tRNA by the magnetic bead method is only applicable for the cell extract of *E. coli*, and must be further optimized for other bacteria or eukaryotes. In general, this method is more practical than most current methods for tRNA removal. The direct quantification by PCR is a valuable tool to assess cell extracts for high-fidelity codon reassignment. Looking forward, this robust platform has the potential to be applied in emerging synthetic biology and biomedical engineering applications.

### 2.2 Depletion of Specific tRNAs

#### 2.2.1 Resin-Bound Colicin D

For specific tRNA removal, the primary issue is codon choice, while excluding Trp and Met with only one encoding codon. According to previous studies, the tRNase colicin D (ColD) can specifically recognize and degrade four different tRNA^Arg^ species of *E. coli*, including the tRNA_ICG_, tRNA_CCG_, tRNA_UCU_, and tRNA_CCU_ (Tomita et al., 2000). This method (Figure 3B) takes full advantage of this property to inactivate all the tRNA^Arg^ from the *E. coli* cell extract (S12) (Kim et al., 2006), resulting in a inability to incorporate Arg in this system. It was further demonstrated that ColD-treated lysates did not affect the ability of protein synthesis, under the condition of supplementing tRNA^Arg^. More importantly, this creates favorable conditions for rearrangements of the remaining Arg codons to expand the genetic codon (Lee et al., 2016). There are two reasons for choosing AGG as the sole codon encoding Arg. Firstly, this is a rare codon, while tRNA^UCU^ and tRNA^CCU^ are relatively resistant to ColD digestion, resulting in a residual amount remaining in the treated S12 lysates. For specific sense codon rearrangement, it is also important to take into account the flexible effect of wobble base pairs in the *in vivo* decoding process (Das and Duncan Lyngdoh, 2014). Although this method is simple and feasible in reconstructing translation systems, it can only be used to rearrange Arg codons and not for other types of tRNAs. Therefore, there are more other tRNases to be explored for use in similar approaches, such as the tRNA^Lys^ anticodon nuclease PrrC (Blanga-Kanfi, 2006). Overall, this method expands further implementations for building a platform for a small number of sense codon rearrangements, and it creatively broadens new horizons and ideas for reconstructing a more general platform.

#### 2.2.2 DNA-Hybridization Chromatography

Emerging attempts have been made to free up more sense codons for the incorporation of multiple ncAAs in the genetic codon simultaneously. Alexandrov et al. demonstrated that isoacceptor tRNAs can be removed through DNA-hybridization chromatography of the standard *E. coli* S30 lysate (Cui et al., 2017). For this approach (Figure 3B), DNA oligos with 3′-amine modifications were designed complementary to the sequence spanning the D-arm down to the anticodon loop of the targeted native isoacceptor tRNA (tRNA^Arg^_UCU_), and were immobilized on an NHS-activated matrix. Then, the treated DNA oligos are mixed with the extract to carry out the hybridization reaction for the chromatographic depletion. The authors introduced a novel criterion for tRNA removal efficiency as follows:

$$\mathrm{tRNA}\;\mathrm{removal}\;\mathrm{efficiency}=1-\left(\frac{\mathrm{RFU}\left(\mathrm{Depleted tRNA}\right)}{\mathrm{RFU}\left(\mathrm{Depleted tRNA}+\mathrm{t7 tRNA}\right)}\right)$$

According to this formula, there are many factors contributing to the more precisely quantified tRNA removal. First of all, the feasibility of this method was demonstrated since the protein expression level could be restored with the addition of the targeted synthetic tRNA in the tRNA-depleted lysate. The depletion efficiency is also related to the number of corresponding decoded codons contained in the template of protein synthesis. For example, using super-folder green fluorescent protein (sfGFP) as the template, the deletion rates of one and six AGG codons were nearly 100 and 90%, respectively. Therefore, the depletion efficiency is determined depending on codon composition in different templates, which further illustrates the role of a specific tRNA and the ability of protein synthesis. Early studies identified a biased distribution of tRNA abundance for bacteria growing at different rates (Dong et al., 1996). This could directly affect the quality of the prepared cell lysate. To ensure basic levels of protein synthesis, several new technical means can be used to accurately measure the amount of specific tRNAs to determine the optimized time for collection of bacteria, such as the modification-induced misincorporation tRNA sequencing (mim-tRNAseq) method (Behrens et al., 2021).

Subsequently, several implemented effective measures were taken to abrogate the targeted tRNA activity and liberate the corresponding sense codons (Cui et al., 2018). This strategy offers several improvements over the above-mentioned methods. Firstly, the antisense oligonucleotides are not affected by the high-level complex molecular structures in tRNAs that reform the L-shaped conformation. Secondly, 2′O-Me modifications of the antisense oligonucleotide perform better than other types in the entropic stabilization to the hybrid base pairs (Yildirim et al., 2014). It should be noted that methylated oligonucleotides display slow dissociation kinetics from the target tRNA, resulting in better RNA strand displacement. This is particularly important for the designed sequence targeting a specific tRNA in the *E. coli* S30 lysate. The hybridization ability shows inconsistencies in the sequence of the same tRNAs between species (*E. coli* and *Leishmania tarentolae*), even though they are located in the anticodon or variable loop region in the same cell. A few heterologous factors need to be further explored to improve the general applicability of the method, such as the input ratio of tRNA and oligonucleotides, incubation time, and temperature, which requires further work in the future. In addition, the two-step protein synthesis reaction system was divided into the treated extract and the total RNA. The described standard *L. tarentolae* extract (LTE) (Mureev et al., 2009) was generated by removing the total RNA by the ethanolamine-sepharose column method. The total RNA was isolated from the lysates. Then, individual isoacceptor tRNAs were selectively sequestered from the purified total RNA by the treatment with DNA oligos with 2′O-Me modifications. Finally, the above two parts are added to the whole reaction system to minimize the background level. Although a somewhat cumbersome, this method is worth learning and considering to reduce the background activity. Treatment with the RNase-related compound angiogenin (ANG) (Su et al., 2019), which can cleave tRNA anticodons to inactivate specific codons, may be a feasible strategy for freeing up sense codons. More effective strategies for the tRNA denaturation approach are expected to be developed in the near future.

## 3 Specific Purification of Individual tRNAs

To construct a different reduced-codon set to support protein translation, the source of tRNA preparation and tuning the composition of the tRNA pool is critical. Recent studies have demonstrated various methods for the preparation of tRNAs for *in vitro* translation systems. Nevertheless, current methods for the isolation of specific tRNAs from host cells involve complicated procedures. In the following section, we discuss the growing research on the preparation of individual tRNAs *in vivo* and *in vitro*, with an emphasis on three methods *in vivo* (Table 1).

**TABLE 1:** Advantages and disadvantages of different tRNA production methods.

Approach	Method	Advantages	Disadvantages	References
Purification *in vivo*	Hydrophobic tagging	Modified nucleotides, low cost, simple operation	Multiple complicated procedures, cannot distinguish isoacceptor tRNAs	Kothe et al. (2006)
	DNA probe-elution	Cost-effective, modified nucleotides, simple operation	Low yield, low purity, low binding efficiency	Kaneko et al. (2003)
	DNA probe-digestion	High purity, high specificity, well-established method	Extensive downstream purification, using toxic reagents	Nilsen (2013)
*In vitro* biosynthesis	Direct	Easy purification, fast, the mutagenesis of the tRNA is easy	Addition of nucleotides at the 3′end, the first base must be a guanine, without modified nucleotides	Li et al. (1999)
	Hammerhead	Fully active in aminoacylation; Not limited to the first base	No 5′phosphorylation, without modified nucleotides, addition of nucleotides at the 3′ end	Fechter et al. (1998)
	RNase P	Not limited to the first base; no addition of nucleotides at the 3′ end	Without modified nucleotides	Hibi et al. (2020)
Chemical synthesis	Solid-phase chemical synthesis	Modifications possible, easy purification, fast, no sequence-specific optimization	Expensive equipment required, length limited, limited availability of labeled or modified phosphoramidites	Ogilvie et al. (1988)

### 3.1 *In vivo*

**FIGURE 4:**
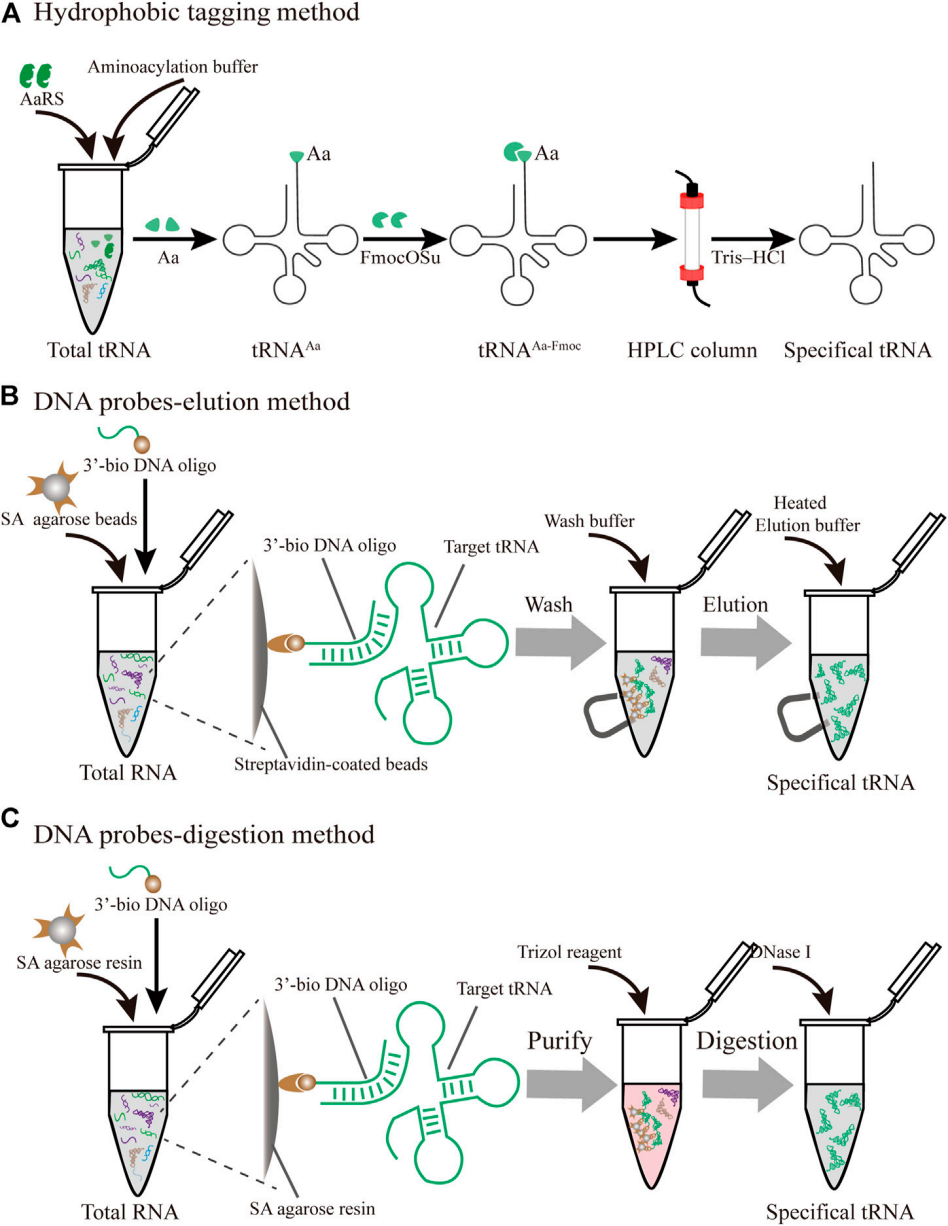
Different strategies for the purification of specific individual tRNAs produced *in vivo* **(A)** Hydrophobic tagging method. The hydrophobic tag (FmocOSu) can react with the free amino group on the aminoacylated tRNA, which can be separated from other tRNAs due to its high molecular weight, then purified and de-acylated **(B)** DNA probe-elution method. Biotinylated DNA oligonucleotides are immobilized on streptavidin sepharose beads, and individual tRNAs are isolated from total RNA using a magnet **(C)** DNA probe-digestion method. The hybridized tRNAs-oligo complex was purified from the tRNAs-oligo-resin complex using Trizol. Individual tRNAs can be released using DNase I

The posttranscriptional modifications of tRNAs play vital roles in the accurate decoding of mRNAs inside the ribosome (Agris et al., 2017). In particular, the tRNA anticodon domain (positions 34 and 37) contains various specific modifications that facilitate the recognition of cognate and wobble codons, which directly influence the translational fidelity (Pereira et al., 2018). Although intracellular purification remains a challenge, there are several novel advances in the purification of specific tRNAs produced *in vivo* (Figure 4).

#### 3.1.1 Hydrophobic Tagging Method

In protein translation systems, the requirement for purifying individual tRNAs *in vivo* has become increasingly prominent. A prominent approach relies on the introduction of a hydrophobic tag for the specifically charged aminoacyl-tRNAs (aa-tRNA) followed by hydrophobic interaction chromatography (Figure 4A) (Kothe et al., 2006). The used hydrophobic tag was 9-fluorenylmethyl-succinimidyl carbonate (FmocOSu), which can react with the free amino group of a selected aa-tRNA. Therefore, the hydrophobicity of this complex is significantly increased compared with other tRNAs, which in turn increases the retention time in the chromatographic column, and finally results in its isolation from total tRNA. Although the used raw material is available at a low cost, and the operation is relatively simple, the entire purification process requires multiple steps, including aminoacylation, modification, purification, and deacylation. In addition, this hydrophobic tagging method has broader applicability for diverse tRNAs than previous approaches (Cayama et al., 2000), because it solely depends on the specific aa-tRNA synthetases and the specific amino acids. The aaRS can only aminoacylate tRNA with one specific amino acid, but it cannot recognize and distinguish the isoacceptor tRNAs. According to this principle, the purified species are a complex of tRNAs encoding the same amino acid. This method is therefore not suitable for the fine-tuning of transfer RNA (tRNAs) in the cell-free lysate. Recent research found that a two-dimensional liquid chromatography (2D-LC) integrating a weak anion-exchange method could be used to isolate tRNA^Val^_UAC_ and tRNA^Leu^_CAG_ (Cao et al., 2020). These purified individual tRNAs might be applied to the minimal codon protein synthesis system. In terms of the cost and feasibility of purification, these methods will open new avenues in the process of simplified chromatographic purification.

#### 3.1.2 DNA Probe-Elution Method

In an early study on probe-elution, biotinylated DNA oligonucleotides were immobilized onto streptavidin agarose beads to isolate individual tRNAs from the *Leishmania tarentolae* (Kaneko et al., 2003). Recently, Söll et al. incubated 5′biotinylated oligo-beads with yeast tRNAs in the equilibrated buffer, and this complex was washed several times to remove the nonspecifically bound or loosely bound molecules. The pure tRNA_m_^Glu^ was eluted by a low-salt buffer at high temperature (Rinehart et al., 2005). Although the principle and operation of this procedure are relatively simple, verification of the tRNAs from a cell by northern blot may be necessary (Figure 4B). However, the current purified volume and purity are relatively low for the distribution and regulation of different species of tRNA for *in vitro* protein synthesis, which hinders its application and development. The following different factors are worth exploring to produce high-quality tRNA. First of all, the individual tRNAs could be over-expressed by constructing recombinant plasmids with a strong constitutive *E. coli* promoter (Cervettini et al., 2020). The main purpose of this process is to enrich enough tRNA for initial extraction and purification, greatly reducing the proportion of non-target tRNAs. In addition, the combination of a chromatographic spin column and streptavidin-coated agarose beads may have more obvious advantages in improving the binding and purification efficiency for bulk purification. The continuous circulation process in the Chaplet Column Chromatography (CCC) method circumvents these drawbacks to a certain extent (Suzuki and Suzuki, 2007). Similarly, the choice of synthetic 3′-biotinylated DNA probes and chromatographic columns can be changed according to the amount of the *Bos taurus* mitochondrial tRNA mixture, exhibiting great flexibility and scalability.

#### 3.1.3 DNA Probe-Digestion Method

With easy availability, biotinylated DNA oligonucleotide probes complementary to the targeted tRNA were successfully used to extract the tRNA-derived stress-induced RNAs (tiRNAs) fraction by the gel purification process (Nilsen, 2013). The hybridized tiRNA-oligo complex was pulled down using streptavidin agarose beads (Figure 4C). With the addition of the Trizol reagent, the targeted tRNA fraction can be freed *via* digestion with DNase I (Akiyama et al., 2020). While initially met with some skepticism, this approach was developed into feasible methods for the isolation of endogenous mature tRNAs, such as tRNA^Ala^_AGC_ and tRNA^Gly^_GCC_. Since tRNAs have a complex molecular structure and are heavily modified, this approach conquered the current limitation of tRNA purification from host cells and opened up a new Frontier in tRNA biology. Nevertheless, several disadvantages of this approach should be addressed. To obtain high-purity target tRNA, toxic Trizol was used repeatedly throughout the purification process, which should be circumvented by seeking alternative reagents. The designed sequences of DNA probes need to be optimized due to the high sequence similarity between the selected tRNA and its isoacceptor tRNAs. Finally, there is a lack of scientific evaluation criteria for the purity and activity of purified tRNA. Emerging technologies, including high-throughput tRNA sequencing (Shigematsu et al., 2017; Erber et al., 2020) and immuno-northern blotting (Mishima et al., 2015; Mishima and Abe, 2019), may be applied to push forward tRNA purification technology.

### 3.2 *In vitro*

**FIGURE 5:**
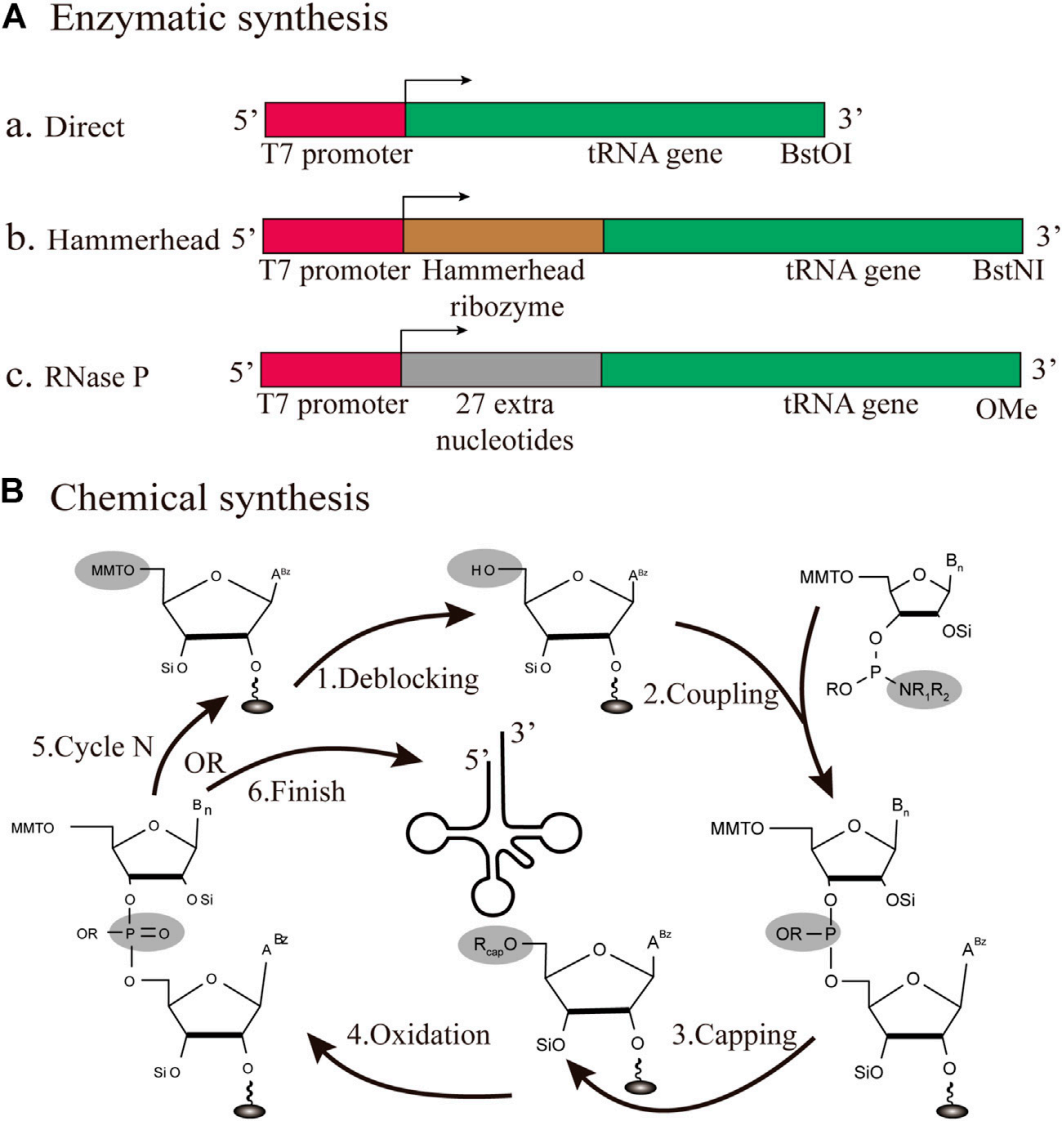
Different strategies for the production of individual tRNAs *in vitro* **(A)** Enzymatic synthesis. Three different patterns for tRNA transcription are based on the T7RNP transcription process, including the direct pattern, hammerhead pattern and RNase P pattern **(B)** Chemical synthesis. The production of tRNA is based on solid-phase chemical synthesis, with each cycle encompassing de-blocking, coupling, capping, and oxidation.

In this section, we will provide a summary of *in vitro* tRNA production methods, mainly including enzymatic and chemical synthesis (Figure 5). This part aims to discuss several crucial points of the tRNA preparation literature, including the latest advances.

#### 3.2.4 Enzymatic Synthesis

Among bacteriophages T3, T7, and SP6, T7 RNA polymerase (T7RNP) offers the largest yield of RNA transcription. Since the length of tRNA is between 75 and 95 nt, the T7RNP transcriptional synthesis of small RNA has been established in initial reports (Milligan and Uhlenbeck, 1989). Originally, there were two major flaws in the T7 transcription process. One defect is that its transcription efficiency mainly depends on the specific recognition of its cognate promoter sequence, incorporating an unfavorable sequence at the 5′-end. On the other hand, the runoff of T7 transcription results in a mixture of products with 3′-end heterogeneity, which usually results in multiple consecutive A nucleotides at the end of the product (Helm et al., 1999). Three different transcription patterns for tRNA transcription will also be discussed based on their applications in the field of transcriptional product heterogeneity (Figure 5A). In a direct pattern, the transcription template starts with the T7 promoter sequence, followed by the encoded tRNA gene sequence, which is directly transcribed (Li et al., 1999). This approach works for most mature tRNAs within cells, but it is limited to tRNA sequences that do not begin with a G, such as tRNA^Asn^ and tRNA^Pro^. The ribozyme pattern is a fusion between the T7 promoter sequence and the tRNA gene, encoding a ribozyme called hammerhead, which has a cis-acting, self-cleavage ability (Huang et al., 2019). This design intends to circumvent the above-mentioned deficits and release a tRNA transcript with the desired 5′- sequence (Korencic et al., 2002). The main advantage of this approach is that pure transcripts can be obtained directly in large quantities for all tRNAs. Although the 5′-OH tRNA transcript can be aminoacylated in this reaction, they proved to be active. Additionally, RNase P can catalyze tRNA maturation with the generation of tRNAs with homogeneous 3′and 5′ends (Fukunaga et al., 2006). RNase P has a catalytic RNA subunit, which can cleave homogeneous 3′-OH ends in tRNAs of interest (Gossringer et al., 2012). The 2′-methoxy modification of the second nucleotide 5′end at the primer prevents additional nucleotide amplification of the 3′-terminal transcripts. Therefore, the latest research fully integrated the above two characteristics, to achieve the production of transcripts of high quality (Hibi et al., 2020). Using this approach, 21 different tRNAs from *E. coli* were analyzed by urea-PAGE, and the homogeneity of the major synthesis was successfully verified. What’s more, the purified iVTtRNA can be sufficiently aminoacylated by the corresponding aaRS. This breakthrough demonstrates that despite lacking any modifications *in vivo*, tRNA transcribed using RNase P was capable of protein synthesis comparable to the native tRNA mixture. The *in vitro* transcribed transfer RNA (iVTtRNA) lacks the modifications at position 37, which possibly allow undesirable wobbling at codon first positions and frameshifts. Indeed, generating the complex base modifications in the anticodon loop regions of iVTtRNA will be an important perspective in decoding accuracy required for the appropriate modification enzymes like the TsaD and TrmD modification enzymes. The introduction of modified nucleotides into the anticodon loop of iVTtRNAs shows a significant increase in the aminoacylation, the protein yield, and the fidelity. Overall, this is a fast, inexpensive and efficient method for the production of specific tRNAs of good purity, providing favorable conditions for *in vitro* protein synthesis based on the iVTtRNA system.

#### 3.2.5 Chemical Synthesis

In early studies, solid-phase chemical synthesis was used for the production of 77 nt tRNA^Met^ (Ogilvie et al., 1988). The whole production cycle involves de-blocking, coupling, capping, and oxidation (Figure 5B). According to the sequence of chemical synthesis from the 3′- to the 5′-terminus, the synthetic target tRNA is finally obtained through reversed-phase high-performance liquid chromatography (HPLC) purification (Baronti et al., 2018). From the cost perspective, the upper limit of the synthesizable length is approximately 80 nt. In this approach, the synthesis mainly depends on the used phosphoramidite monomers and the spatial structure of target sequences. Accordingly, synthetic production is a challenge for all tRNAs, which are generally structurally complex molecules. However, it is still feasible and effective to adopt chemical methods for the synthesis of tRNAs with specifically modified bases (Vare et al., 2017). Chemical synthesis has been used for decades in the production of small RNAs, and it still plays a major role. For future studies, there is an urgent need to develop a lower-cost, high-efficiency protocol, focusing on the manipulation of tRNAs.

## 4 Simplified Codon Protein Synthesis With tRNA Complement

**FIGURE 6:**
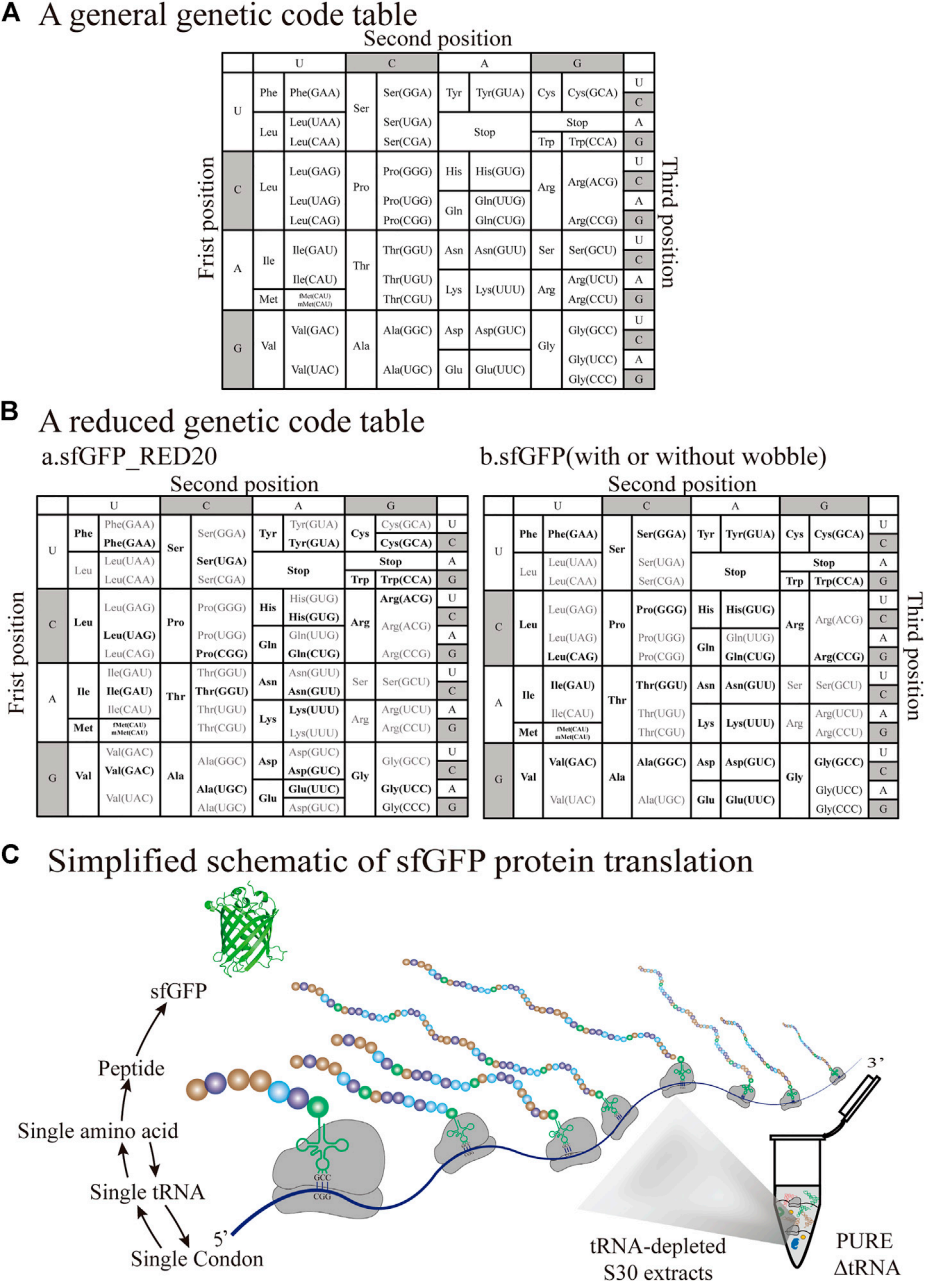
Selection of different tRNA sets for the decoding of sfGFP templates with reduced-codons **(A)** The native genetic code table of *E. coli* **(B)** Two reduced genetic code tables for the production of different versions of sfGFP (sfGFP_RED20 and sfGFP; with or without wobble, respectively, Unused codons are represented in gray, while frequently used codons are represented in black **(C)** Simplified schematic of sfGFP protein translation based on the tRNA-depleted S30 extract and PURE ΔtRNA system. This sfGFP mRNA clearly shows the one-to-one correspondence in the codon-tRNA-amino acid relationship.

The general codon table is a genetic coding rule shared by almost all organisms (Figure 6A). Since the 20 common proteinogenic amino acids are decoded by 61 codons (triplets), there is a great redundancy in the standard code. To achieve protein synthesis with reduced codons, two major factors need to be considered in a cell-free system. The first factor is to exclude the influence of endogenous tRNAs in the extract, so it is necessary to establish a cell-free system with completely removed native tRNAs. The second factor is the synthesis and purification method of added tRNA for the manipulation, so it is necessary to seek an alternative way for the synthesis of tRNA. Generally, these two conditions have been discussed above. Numerous attempts to produce different proteins by reducing codon redundancy have been published. Here we present an overview of the designed pattern of different protein synthesis approaches based on the tRNA-depleted S30 extract and PURE ΔtRNA system (Table 2). Although the complete removal of endogenous tRNAs is currently still a challenge, some of them have important reference value and milestone significance in the application of simplified and minimal codon synthesis.

**TABLE 2:** A reduced set of tRNA used for protein synthesis based on the cell free system.

Amino Acids	Codons	tRNAs	Product	tRNA source	Reaction system	References
11	11	11	RGS peptide	*in vitro* transcribed	tRNA-depleted S30 extracts	Cui et al. (2015)
20	45	41	sfGFP_T1			
20	30	26	sfGFP_T2			
20	44	39	sfGFP_T3			
23	23	32	P9-Naa peptide	*in vitro* transcribed	PURE ΔtRNA	Iwane et al. (2016)
2	2	2	(Val)40	*in vitro* transcribed	tRNA-depleted S30 extracts	Salehi et al. (2017)
20	20	21	sfGFP-RED20	chemically synthesized	PURE ΔtRNA	Calles et al. (2019)
20	20	21	sfGFP	*in vitro* transcribed	PURE ΔtRNA	Hibi et al. (2020)

### 4.1 tRNA-Depleted S30 Extracts

This novel semisynthetic tRNA complement system was primarily used for reassigning sense codons in protein synthesis based on the depleted endogenous tRNAs of BL21 (DE3)GOLD S30 cell extract (Cui et al., 2015). The *in vitro* peptide expression assay takes advantage of synthesizing a short peptide reporter named the RGS-peptide (peptide RGSIDTWV) (Stein and Alexandrov, 2014). An advantage of this method is that it not only reduces the pressure of the amount of codons used in the expression template, but also efficiently evaluates the efficiency of iVTtRNA decoding. This approach provides a simple and efficient alternative to the preparation of purified tRNA for verification of aminoacylation activity, without the complex process of radiolabeling employed previously (Schwartz and Pan, 2017). Importantly, it was found that 17 amino acids can be efficiently decoded by the corresponding iVTtRNAs, with the exception of Glu, Asn, and Ile. Therefore, a tRNA mixture containing 20 natural amino acids was constructed by combining *in vivo* purification with an *in vitro* transcription strategy. What’s more, sfGFP templates containing changed sets of codons (Pedelacq et al., 2006) were used to confirm that the tRNA mixture retains the decoding ability of the synthesized full-length protein, as expected. It is worth noting that different versions of the sfGFP gene have been simplified to free up more sense codons. At present, the least tested is the use of 26 different tRNAs and 30 amino acids with the template sfGFP_T2 (Figure 6B). Although the complete removal of native tRNAs remains challenging, this approach has broken the existing bottleneck for the redundancy of the general genetic codon, significantly broadening the artificially designed platform for protein synthesis using the smallest number of codons. However, recent work has taken advantage of the real-time PCR method for the accurate quantitative analysis of the remaining amount of tRNA in the treated cell extract, rather than relying solely on protein synthesis ratios (Salehi et al., 2017). A synthetic tRNA^fMet^ and tRNA^Val^ were successfully used to produce a polypeptide named (Val)_40_, achieving artificially the one-to-one correspondence between the codon-tRNA-amino acid relationship. Although there are only two amino acids in this polypeptide, the feasibility of this method has been preliminarily verified, which provides the conditions for the synthesis of macromolecular proteins. It is clear that residual tRNAs substantially decrease the codon reassignment efficacy of macromolecular protein synthesis. As mentioned above, advances in this direction have been made in the evaluation and optimization of the complete deletion of the native tRNA mixture to simplify the genetic codon.

### 4.2 PURE ΔtRNA

The reconstituted PURE system (PURE ΔtRNA), which lacks all native tRNAs, is an appropriate system for the manipulation of different tRNA sets for protein expression. The introduction of a new concept of artificial codon-box division is based on blanking a sense codon, which is then reassigned to a non-natural amino acid (Iwane et al., 2016). In this methodology, the flexible *in vitro* translation (FIT) system (Hipolito and Suga, 2012), which contains only 32 *in vitro* tRNA transcripts, was referred to as the FIT-32t system. Using this FIT-32t system, the authors synthesized the P9-Naa peptide, which contained the 20 natural amino acids and three non-natural amino acids (^Ac^K, ^Iodo^F, and Cit), which was demonstrated to be the major product by MALDI-TOF-MS. Although the relative yield of this P9-Naa peptide was approximately 15% of that obtained using the native tRNA mixture, it demonstrated the feasibility of reducing the redundancy of the genetic codons. In addition, the established RED20 code system is defined that the map containing the 20 sense codons corresponding to 20 amino acids with the addition of the three stop codons (Calles et al., 2019). In this sfGFP-RED20 template system, there are significant differences in the total protein-expression capacity between the 21 synthetic tRNAs and the full set of natural tRNAs. This difference may be caused by many different factors, such as a lack of modification on the synthetic tRNAs, which affects their ability to decode the mRNA, as well as the frequency of codon usage or the promiscuous decoding of null codons, among others. This reduced set of tRNAs encoding RED20 will play a foundational role in reassigning sense codons and simplifying the general codon box. Consistent with this approach, the developed 21 iVTtRNAs without nucleotide modifications were able to restore the sfGFP and dihydrofolic acid reductase (DHFR) production capacity based on the PURE system (Hibi et al., 2020). In consideration of the effect of wobble base pairings, an sfGFP template with or without wobble bases was designed to demonstrate the fidelity of protein translation using this system (Figure 6B). A single aminoacyl-tRNA can decode multiple codons since “wobble” base pairs in natural translation system. Therefore, the genetic codon box cannot be simply divided out of the blank codons for the rearrangement of novel amino acids. Due to the wobble base pair, the designed any set of 20 tRNA species can cover more than 20 codons, leaving significantly less than 41 codons to novel amino acids. An ideal approach would be to artificially assign each codon to a specific tRNA and a specific amino acid to achieve a one-to-one correspondence (Figure 6C). This feature is not applicable in natural organisms due to biological and artificial isolation phenomena. This artificial design pattern provides a powerful platform for the exploration of biosafety issues (Schmidt, 2019). However, a new direction of these redesigned codon systems should be explored in the future. Firstly, it is worthwhile to construct a universal database that optimizes the best tRNA set for different functional proteins to achieve the highest possible yield and efficiency. Furthermore, ensuring the fidelity of the translated opened reading frame is necessarily important in the protein translation process. It is well known that particular codons and sequence contexts are “shifty” and frequently cause frameshifts. This follows that a particular set of 20 codons would better maintain the reading frame than other possible sets. The iVTtRNA transcription and tRNA modification enzymes need to be coupled to maintain the fidelity of decoding. Finally, advances have been made in the incorporation of multiple non-natural amino acids and the construction of a minimal cell system.

## 5 Conclusions and Prospects

Simplified codon protein synthesis with tRNA complement has unique advantages, which were summarized and discussed in this review. To obtain the tRNA-depleted S30 extract, several approaches have been developed to remove the native tRNA set, including the ethanolamine-sepharose column and RNase-coated magnetic bead methods. In addition, further methods for the depletion of specific tRNAs from S30 lysates were also generalized, including the enzymatic depletion method and DNA-hybridization chromatography. The advantages and disadvantages of these methods have been summarized and discussed in detail above. At the same time, the preparation of tRNA is still a very important factor, and this paper also comprehensively summarizes *in vivo* and *in vitro* approaches for targeted tRNA synthesis. The strategies of hydrophobic labeling, DNA probe-elution, and DNA probe-digestion were mainly summarized in the section on *in vivo* purification. These approaches can be used to purify a specific tRNA and have modification advantages compared with *in vitro* purification. What’s more, compared with *in vivo* purification and chemical synthesis, the T7RNP-based method for enzymatic tRNA synthesis has many advantages, such as simple operation, low cost, and high yield. Overall, the use of a combination of *in vivo* purification and T7RNP-based enzymatic purification may be the most efficient method for tRNA purification applications. Finally, the key issues and challenges in the tRNA-depleted S30 extract and PURE ΔtRNA systems for different versions of the existing artificially designed genetic codon tables were discussed individually. By emphasizing these crucial elements for simplified codon protein synthesis in a cell-free protein system, this review is intended to provide current progress and explore new challenges in the field of *in vitro* synthetic biology.

Looking forward, this emphasized tRNA complement with protein synthesis system has the potential to be applied in various emerging fields, such as the construction of minimal cells (Noireaux et al., 2011; Stano and Luisi, 2013; Yue et al., 2019), the employment of microfluidic devices (Damiati et al., 2018; Weiss et al., 2018), the incorporation of multiple different ncAAs (Zheng et al., 2018; Chemla et al., 2019; Italia et al., 2019), the direct preparation of bio-conjugates (Bundy et al., 2008; Zimmerman et al., 2014) and other novel biotechnology applications. Aiming at controllably reconstituting a minimum set of compounds, the simplified genetic codon with the CFPS system will play a significant role in the bottom-up assembly of minimal cells. In particular, several efforts have been devoted to making breakthroughs in redesigning genomes by freeing up more sense codons. For instance, the genomic reassignment of the 57-codon *E. coli* MDS42 (*rE.coli-57*) was a major attempt at constructing a fully recoded organism (Ostrov et al., 2016). In this *rE.coli-57*, a total of seven selected codons were replaced with their synonymous codons on a genome-wide scale to expand the genetic codon. However, only four orthogonal nnAAs with novel chemical properties could be incorporated into the targeted protein. By contrast, the Syn61 with a 61-codon genome has a complete genome-wide deletion of the three target codons (amber-TAG, S-TCG, and S-TCA), with their defined synonymous substitution (Fredens et al., 2019). It was demonstrated that the reassignment of vacant TCG codons provides the possibility of further codon expansion. Despite the high synthetic cost and cumbersome construction process, this genomic engineering approach could help release sense codons for rearrangement to a certain extent. In future work, comprehensive research coupling the genomically recoded organisms and the CFPS system might be an alternative approach for enabling the recoding of much more sense codons. What’s more, several emerging approaches such as flexizyme aminoacylation (Katoh et al., 2018; Lee et al., 2019) and the evolved orthogonal aaRS/tRNA pairs (Bryson et al., 2017; Willis and Chin, 2018) could be applied to the minimal codon system for the synthesis of non-canonical biopolymers. Taken together, construction of cell-free protein synthesis with manipulating tRNA, which reduces the use of codons and tRNA species, might provide favorable conditions for simplified cellular mimics or self-replicating systems (Van Nies et al., 2018). We firmly believe that further systematic exploration of the minimal codon protein synthesis system will advance the development of synthetic cells and broaden the field of synthetic biology.
